# Emergence of Dengue Virus Serotype 2 Cosmopolitan Genotype, Brazil

**DOI:** 10.3201/eid2808.220550

**Published:** 2022-08

**Authors:** Marta Giovanetti, Luiz Augusto Pereira, Gilberto A. Santiago, Vagner Fonseca, Maria Paquita García Mendoza, Carla de Oliveira, Laise de Moraes, Joilson Xavier, Stephane Tosta, Hegger Fristch, Emerson de Castro Barbosa, Evandra Strazza Rodrigues, Dana Figueroa-Romero, Carlos Padilla-Rojas, Omar Cáceres-Rey, Ana Flávia Mendonça, Fernanda de Bruycker Nogueira, Rivaldo Venancio da Cunha, Ana Maria Bispo de Filippis, Carla Freitas, Cassio Roberto Leonel Peterka, Carlos Frederico Campelo de Albuquerque, Leticia Franco, Jairo Andrés Méndez Rico, Jorge L. Muñoz-Jordán, Vinícius Lemes da Silva, Luiz Carlos Junior Alcantara

**Affiliations:** Fundação Oswaldo Cruz, Rio de Janeiro, Brazil (M. Giovanetti, C. de Oliveira, F. de Bruycker Nogueira, R.V. da Cunha, A.M.B. de Filippis, L.C.J. Alcantara);; Università Campus Bio-Medico di Roma, Rome, Italy (M. Giovanetti);; Universidade Federal de Minas Gerais, Belo Horizonte, Brazil (M. Giovanetti, J. Xavier, S. Tosta, H. Fristch, L.C.J. Alcantara);; Laboratório Central de Saúde Pública Dr. Giovanni Cysneiros, Goiânia, Brazil (L.A. Pereira, A.F. Mendonça, V.L. da Silva);; Centers for Disease Control and Prevention, San Juan, Puerto Rico, USA (G.A. Santiago, J.L. Muñoz-Jordán);; Organização Pan-Americana da Saúde/Organização Mundial da Saúde, Brasília, Brazil (V. Fonseca, C.F.C. de Albuquerque);; Instituto Nacional de Salud, Lima, Peru (M.P. García Mendoza, D. Figueroa Romero, C. Padilla-Rojas, O. Cáceres-Rey);; Fundação Oswaldo Cruz and Universidade Federal da Bahia, Salvador, Brazil (L. de Moraes);; Fundação Ezequiel Dias, Belo Horizonte (E. de Castro Barbosa);; University of São Paulo, Ribeirão Preto, Brazil (E.S. Rodrigues);; Ministério da Saúde, Brasília (C. Freitas, C.R.L. Peterka);; Pan American Health Organization–World Health Organization, Washington, DC, USA (L. Franco, J.A.M. Rico)

**Keywords:** dengue virus, viruses, zoonoses, vector-borne infections, dengue, cosmopolitan genotype, genomic monitoring, Brazil, South America

## Abstract

We used nanopore sequencing and phylogenetic analyses to identify a cosmopolitan genotype of dengue virus serotype 2 that was isolated from a 56-year-old male patient from the state of Goiás in Brazil. The emergence of a cosmopolitan genotype in Brazil will require risk assessment and surveillance to reduce epidemic potential.

Dengue virus (DENV) is a single-stranded, positive-sense RNA virus that has a genome consisting of ≈11 kb. DENV belongs to the Flaviviridae family (genus *Flavivirus*) and is transmitted by *Aedes aegypti* and *Ae. albopictus* mosquitoes (*1*). DENV has caused a substantial global economic and public health burden and numerous mild to severe epidemics in the Americas, particularly during recent decades (*1*). DENV can be divided into 4 antigenically distinct serotypes (DENV-1–4), which have an interserotype nucleotide variability of ≈30% (*2*). Each serotype is further subdivided into phylogenetically distinct genotypes often named according to their geographic origin, even though some DENV serotypes have spread to other regions (*2*). According to epidemiologic reports, recent dengue epidemics in Brazil and South America were mainly driven by the circulation of DENV-1 and DENV-2 serotypes (*3*,*4*). DENV-2 contributed substantially to dengue-related mortality in the region. 

DENV-2 includes 5 distinct nonsylvatic genotypes. Circulation of the Asian I and II genotypes (also known as DENV-2 genotype IV) is mostly circumscribed to Asia. The Asian–American genotype, also known as the Southeast Asian–American or genotype III, replaced the American genotype (DENV-2 genotype I) in the 1980s (*5*). The cosmopolitan genotype (DENV-2 genotype II) is the most widespread and genetically heterogeneous genotype (*6*). This genotype is circulating in Asia, the Middle East, the Pacific Islands, and Africa and contributes substantially to the global dengue burden (*6*). The global dispersal of this genotype might have driven extensive intragenotypic diversity, potentially favoring widespread expansion (*6*).

Cosmopolitan lineages continue to expand geographically, and recent introductions have been reported in Asia and Africa (*7*,*8*). In South America, the cosmopolitan genotype was detected in Peru in 2019 and spread mainly in Madre de Dios Province, where 4,893 total dengue cases were reported during that year (*9*). However, much is still unknown about its genomic diversity, evolution, and transmission dynamics in the region. Because each genotype might result in different clinical outcomes or enhanced virus dispersal, surveillance of circulating strains is pivotal for public health preparedness (*5*).

We report a case of DENV-2 cosmopolitan genotype in Goiás state, a well-connected region located in midwestern Brazil. We combined mobile genomic sequencing and phylogenetic data to provide preliminary insight regarding the transmission dynamics of this genotype in Brazil. 

The patient was a health worker at the Control Center and Zoonosis in Aparecida de Goiânia, located in Goiás, who had no travel history. The patient had symptoms (fever, myalgia, nausea, retroorbital pain, back pain, headache) compatible with an arbovirus infection on November 26, 2021. A serum sample was collected and sent to the Central Public Health Laboratory of Goiás for molecular screening. Viral RNA was extracted by using the QIAmp Viral RNA Mini Kit (QIAGEN, https://www.qiagen.com) and tested by quantitative reverse transcription PCR for arboviruses, including Zika, chikungunya, and yellow fever viruses and DENV-1–4. Molecular testing confirmed DENV-2 infection. We performed genome sequencing by using nanopore technology to rapidly identify the DENV genotype as part of an active arboviral real-time monitoring effort in collaboration with public health laboratories in Brazil (Appendix). 

We performed phylogenetic analysis by using the DENV Typing Tool (Genome Detective, http://genomedetective.com), which consistently placed the strain from Brazil in a clade within the cosmopolitan lineage and showed maximum statistical bootstrap support (100%) (Appendix Figure 1). Time-resolved maximum-likelihood trees demonstrated that the isolate obtained in this study clustered with 2 recently described DENV-2 strains isolated in Peru in 2019 (bootstrap support, 96%) (Figure; Appendix Figure 2), suggesting a possible cross-border transmission. This cluster in South America diverged from strains observed in Bangladesh that were collected during 2017–2019 (bootstrap support 100%), suggesting a complex transmission scenario mediated by transcontinental travel (Figure; Appendix Figure 3). 

**Figure:**
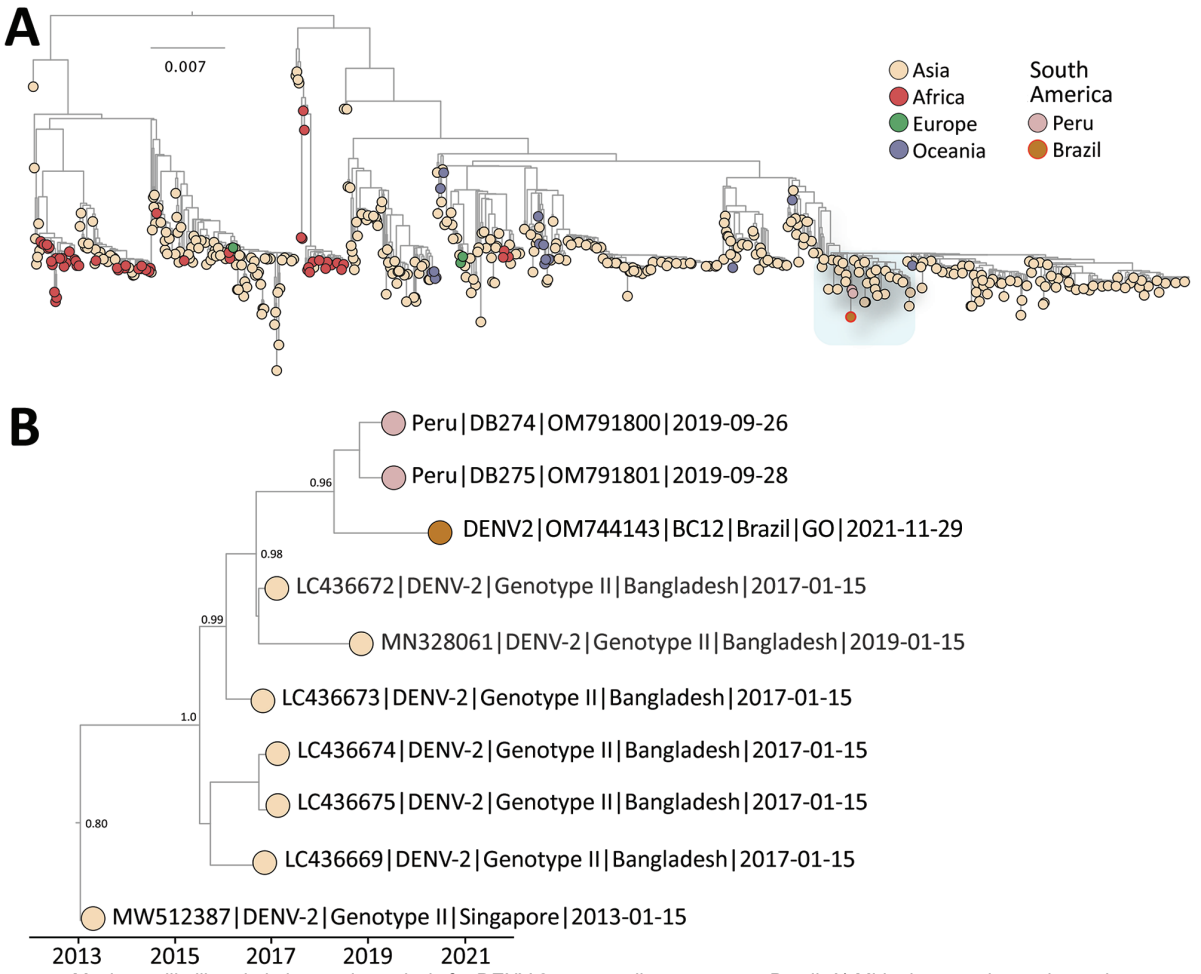
Maximum-likelihood phylogenetic analysis for DENV-2 cosmopolitan genotype, Brazil. A) Midpoint rooted tree shows the evolutionary relationships of the complete genome sequence from the DENV-2 cosmopolitan genotype identified from a patient in Goiás state, Brazil (orange circle), and 1,089 publicly available sequences from GenBank. Scale bar indicates nucleotide substitutions per site. Colors represent different sampling locations. Blue highlighting shows area enlarged in panel B. B) Time-resolved maximum-likelihood tree showing the blue highlighted area from the larger tree in panel A. Colors indicate geographic location of sampling. Support for branching structure is shown by bootstrap values at key nodes. DENV-2, dengue virus serotype 2.

In summary, although genetic data alone cannot determine transmission directionality, phylogenetic analyses indicated that the DENV-2 cosmopolitan genotype sequence recovered from Goiás clustered with strains isolated in Peru, which deviated from a robust clade of sequences isolated in Bangladesh during 2017–2019 (Appendix Table). Brazil will need to improve DENV screening and sequencing to determine whether the virus is endemic or represents a recent introduction from elsewhere, such as Peru and Asia. The emergence of a DENV-2 cosmopolitan genotype in Brazil will require active outbreak risk assessment to reduce epidemic potential. Considering the potential for spread in this region, we advocate for a shift to active surveillance to ensure adequate control of any potential outbreak of this genotype across South America.

Appendix Additional information for emergence of dengue virus serotype 2 cosmopolitan genotype, Brazil.
