# Supplementary material for: Emergence of Dengue Virus Serotype 2 Cosmopolitan Genotype, Brazil
Source: Emerg Infect Dis. 2022 Aug;28(8):1725–7. doi: 10.3201/eid2808.220550 (PMC9328905; doi:10.3201/eid2808.220550)
Supplement: Appendix — Additional information for emergence of dengue virus serotype 2 cosmopolitan genotype, Brazil. [file 22-0550-Techapp-s1.pdf]

Article DOI: https://doi.org/10.3201/eid2808.220550

# Emergence of Dengue Virus Serotype 2 Cosmopolitan Genotype, Brazil

## Appendix

## Materials and Methods

### Ethics Statement

This project was reviewed and approved by the Pan American Health Organization Ethics Review Committee (reference no. PAHO-2016–08–0029) and Research Ethics Committee (Comitê de Ética em Pesquisa, CEP) of Federal University of Minas Gerais, Belo Horizonte, Brazil (CEP no. 32912820.6.1001.5149). Clinical samples are available for research purposes during outbreaks of national concern in accordance with the terms of the National Health Council Resolution no. 510/2016 and National Research Ethics Commission, Ministry of Health, Brazil (CONEP, Comissão Nacional de Ética em Pesquisa, Ministério da Saúde). This resolution authorizes the use of clinical samples collected in the Central Public Health Laboratory, Brazil, without informed consent to accelerate the acquisition of knowledge and contribute to surveillance and outbreak responses. The sample processed in this study was obtained anonymously from excess material after routine diagnosis of arboviruses by a public health laboratory that belongs to the public network within the Ministry of Health, Brazil.

### Sample Collection, Viral RNA Isolation, and PCR Screening

A serum sample was collected for molecular diagnosis and sent to the Central Public Health Laboratory of Goiás for molecular screening. Viral RNA was extracted by using the QIAmp Viral RNA Mini Kit (QIAGEN, https://www.qiagen.com) and tested by quantitative reverse transcription PCR for arboviruses, including Zika virus, chikungunya virus, dengue virus (DENV) serotypes 1–4, and yellow fever virus. Molecular testing confirmed DENV-2 infection.

### Library Preparation and Whole-Genome Sequencing

We prepared cDNA from viral RNA by using the ProtoScript II First Strand cDNA Synthesis Kit (*1*) and conducted multiplex tiling PCR by using Q5 High Fidelity Hot-Start DNA

Polymerase (New England Biolabs, https://www.neb.com) and DENV-2 sequencing primers (*2*,*3*). Amplicons were purified by using 1× AMPure XP Beads, and the PCR product concentrations were measured by using the Qubit dsDNA HS Assay Kit on a Qubit 3.0 fluorimeter (Thermo Fisher Scientific, https://www.thermofisher.com). We prepared a DNA library by using the Ligation Sequencing Kit and Native Barcoding Kit (EXP-NBD104; Oxford Nanopore Technologies, https://nanoporetech.com) (*1*). The DNA concentrations of purified PCR products were measured and normalized before the barcode reactions. The sequencing library was loaded onto a R9.4 flow cell (Oxford Nanopore), and data were collected for up to 6 h. In addition, this study included 2 complete genomes of the DENV-2 cosmopolitan genotype from the 2019 outbreak in Madre de Dios, Peru, that were sequenced by the US Centers for Disease Control and Prevention by using published tiling PCR methods on an Illumina MiSeq platform (GenBank accession nos. OM791800, OM791801, https://www.ncbi.nlm.nih.gov/genbank) (*4*,*5*).

### Generation of Consensus Sequences

We performed base calling on raw files by using Guppy software and barcode demultiplexing by using qcat software. We generated de novo consensus sequence assemblies by using Genome Detective (https://www.genomedetective.com) (*5*). A total of 32,315 mapped reads were obtained with a mean sequencing depth of >1,000× and coverage of >86%. The DENV-2 cosmopolitan genotype was identified. The new DENV-2 sequence obtained in this study was deposited in GenBank (accession no. OM744143).

### Phylogenetic Analysis

We constructed phylogenetic trees to explore the relationships between our sequenced DENV-2 genome and those of other isolated cosmopolitan genotypes. We retrieved 1,087 complete genomic coding sequences of DENV-2 cosmopolitan genotypes from GenBank; these data included the lineage date, country of collection, and the first 2 strains from South America isolated in Peru in 2019. Sequences were aligned by using MAFFT (*6*) and edited by using AliView (*7*). The datasets were assessed for the presence of phylogenetic signals by implementing the likelihood mapping analysis tool in the IQ-TREE 2 software (*8*). A maximum likelihood phylogeny was reconstructed by using IQ-TREE 2 software under the HKY+G4 substitution model (*8*). We inferred time-scaled trees by using TreeTime (*9*).

## References


1. Quick J, Grubaugh ND, Pullan ST, Claro IM, Smith AD, Gangavarapu K, et al. Multiplex PCR method for MinION and Illumina sequencing of Zika and other virus genomes directly from clinical samples. Nat Protoc. 2017;12:1261–76. PubMed https://doi.org/10.1038/nprot.2017.066

2. Hill SC, Neto de Vasconcelos J, Gutierrez BG, Thézé J, Jandondo D, Neto Z, et al. Early genomic detection of cosmopolitan genotype of dengue virus serotype 2, Angola, 2018. Emerg Infect Dis. 2019;25:784–7. PubMed https://doi.org/10.3201/eid2504.180958

3. Adelino TÉR, Giovanetti M, Fonseca V, Xavier J, de Abreu ÁS, do Nascimento VA, et al. Field and classroom initiatives for portable sequence-based monitoring of dengue virus in Brazil. Nat Commun. 2021;12:2296. PubMed https://doi.org/10.1038/s41467-021-22607-0

4. Paquita García M, Padilla C, Figueroa D, Manrique C, Cabezas C. Emergence of the Cosmopolitan genotype of dengue virus serotype 2 (DENV2) in Madre de Dios, Peru, 2019 [in Spanish]. Rev Peru Med Exp Salud Publica. 2022;39:126–8. https://doi.org/10.17843/rpmesp.2022.391.10861

5. Vilsker M, Moosa Y, Nooij S, Fonseca V, Ghysens Y, Dumon K, et al. Genome Detective: an automated system for virus identification from high-throughput sequencing data. Bioinformatics. 2019;35:871–3. PubMed https://doi.org/10.1093/bioinformatics/bty695

6. Katoh K, Standley DM. MAFFT multiple sequence alignment software version 7: improvements in performance and usability. Mol Biol Evol. 2013;30:772–80. PubMed https://doi.org/10.1093/molbev/mst010

7. Larsson A. AliView: a fast and lightweight alignment viewer and editor for large datasets. Bioinformatics. 2014;30:3276–8. PubMed https://doi.org/10.1093/bioinformatics/btu531

8. Minh BQ, Schmidt HA, Chernomor O, Schrempf D, Woodhams MD, von Haeseler A, et al. IQ-TREE 2: new models and efficient methods for phylogenetic inference in the genomic era. Mol Biol Evol. 2020;37:1530–4. PubMed https://doi.org/10.1093/molbev/msaa015

9. Sagulenko P, Puller V, Neher RA. TreeTime: maximum-likelihood phylodynamic analysis. Virus Evol. 2018;4:vex042. PubMed https://doi.org/10.1093/ve/vex042

**Appendix Table.** List of accession numbers, collection dates, and locations for dengue virus serotype 2 cosmopolitan genotypes used to analyze genomic sequences in a study of outbreaks in Brazil*

| Accession no. | Location | Collection date | Accession no. | Location | Collection date |
|---|---|---|---|---|---|
| OM791800 | Peru | 2019–09–26 | MK473386 | Kenya | 2016–01–15 |
| OM791801 | Peru | 2019–09–28 | MK513444 | Singapore | 2015–01–15 |
| OM791802 | Peru | 2018–12–31 | MK543448 | China | 2018–01–15 |
| OM744143 | Brazil | 2021–11–29 | MK543449 | China | 2018–01–15 |
| AB189122 | Indonesia | 1998–01–15 | MK543450 | China | 2018–01–15 |
| AB189123 | Indonesia | 1998–01–15 | MK543471 | China | 2018–01–15 |
| AB189124 | Indonesia | 1998–01–15 | MK543479 | China | 2018–01–15 |
| AF276619 | China | 2000–01–15 | MK564476 | China | 2017–01–15 |
| AF359579 | China | 1999–01–15 | MK564477 | China | 2016–01–15 |
| AY037116 | Australia | 1993–01–15 | MK564478 | China | 2016–01–15 |
| AY776328 | Taiwan | 2004–01–15 | MK564479 | China | 2016–01–15 |
| AY858035 | Indonesia | 2004–01–15 | MK564480 | China | 2016–01–15 |
| AY858036 | Indonesia | 2004–01–15 | MK564481 | China | 2017–01–15 |
| DQ448231 | India | 2001–01–15 | MK564482 | China | 2017–01–15 |
| DQ645540 | Taiwan | 2001–10–31 | MK564483 | China | 2017–01–15 |
| DQ645541 | Taiwan | 2001–11–03 | MK564484 | China | 2018–01–15 |
| DQ645542 | Taiwan | 2001–11–12 | MK564485 | China | 2018–01–15 |
| DQ645543 | Taiwan | 2001–12–03 | MK564486 | China | 2018–01–15 |
| DQ645544 | Taiwan | 2001–12–07 | MK564487 | China | 2018–01–15 |
| DQ645545 | Taiwan | 2002–06–17 | MK564488 | China | 2018–01–15 |
| DQ645546 | Taiwan | 2002–06–24 | MK578531 | China | 2016–01–15 |
| DQ645547 | Taiwan | 2002–07–16 | MK578532 | China | 2016–01–15 |
| DQ645548 | Taiwan | 2002–07–20 | MK578533 | China | 2018–01–15 |
| DQ645549 | Taiwan | 2002–08–18 | MK629884 | South Korea | 2015–01–15 |
| DQ645550 | Taiwan | 2002–08–19 | MK629885 | South Korea | 2015–01–15 |
| DQ645551 | Taiwan | 2002–09–12 | MK629886 | South Korea | 2015–01–15 |
| DQ645552 | Taiwan | 2002–09–13 | MK783189 | China | 2017–01–15 |
| DQ645553 | Taiwan | 2002–10–23 | MK783190 | China | 2018–01–15 |
| DQ645554 | Taiwan | 2002–10–26 | MK783191 | China | 2018–01–15 |
| DQ645555 | Taiwan | 2002–11–01 | MK783192 | China | 2018–01–15 |
| DQ645556 | Taiwan | 2002–11–18 | MK783193 | China | 2018–01–15 |
| EF051521 | China | 2001–01–15 | MK783194 | China | 2018–01–15 |
| EU056810 | Burkina Faso | 1983–01–15 | MK783195 | China | 2018–01–15 |
| EU081177 | Singapore | 2005–01–15 | MK783196 | China | 2018–01–15 |
| EU081178 | Singapore | 2005–01–15 | MK783197 | China | 2018–01–15 |
| EU081179 | Singapore | 2005–01–15 | MK783198 | China | 2018–01–15 |
| EU081180 | Singapore | 2005–01–15 | MK783199 | China | 2018–01–15 |
| EU179857 | Brunei | 2005–01–15 | MK783200 | China | 2017–01–15 |
| EU179858 | Brunei | 2005–01–15 | MK783201 | China | 2018–01–15 |
| EU179859 | Brunei | 2006–01–15 | MK783202 | China | 2018–01–15 |
| EU359009 | China | 2007–01–15 | MK783203 | China | 2018–01–15 |
| EU482640 | Viet Nam | 2006–01–15 | MK783204 | China | 2018–01–15 |
| EU482672 | Viet Nam | 2006–01–15 | MK783205 | China | 2018–01–15 |
| FJ196852 | China | 2001–01–15 | MK783206 | China | 2018–01–15 |
| FJ196853 | China | 2003–01–15 | MK783207 | China | 2018–01–15 |
| FJ196854 | China | 1993–01–15 | MK783208 | China | 2018–01–15 |
| FJ882602 | Sri Lanka | 1996–01–15 | MK783209 | China | 2018–01–15 |
| FJ898454 | India | 2006–01–15 | MK783210 | China | 2018–01–15 |
| GQ252676 | Sri Lanka | 2003–01–15 | MK858096 | India | 2014–01–15 |
| GQ252677 | Sri Lanka | 2004–01–15 | MK858097 | India | 2013–01–15 |
| GQ398258 | Indonesia | 1975–01–15 | MK858098 | India | 2016–01–15 |
| GQ398259 | Indonesia | 1976–01–15 | MK858099 | India | 2016–01–15 |
| GQ398260 | Indonesia | 1976–01–15 | MK858100 | India | 2016–01–15 |
| GQ398261 | Indonesia | 1976–01–15 | MK858101 | India | 2016–01–15 |
| GQ398262 | Indonesia | 1976–01–15 | MK858102 | India | 2016–01–15 |
| GQ398263 | Indonesia | 1975–01–15 | MK858103 | India | 2016–01–15 |
| GQ398264 | Indonesia | 1976–01–15 | MK858104 | India | 2016–01–15 |
| GQ398265 | Singapore | 2008–01–15 | MK858105 | India | 2016–01–15 |
| GQ398266 | Singapore | 2007–01–15 | MK858106 | India | 2016–01–15 |
| GQ398267 | Singapore | 2007–01–15 | MK858107 | India | 2016–01–15 |
| GU131843 | Burkina Faso | 1986–01–15 | MK858108 | India | 2016–01–15 |
| GU370050 | Singapore | 2007–04–01 | MK858109 | India | 2016–01–15 |
| GU370051 | Singapore | 2008–08–01 | MK858110 | India | 2016–01–15 |
| HM488257 | Guam | 2001–01–15 | MK858111 | India | 2016–01–15 |
| JE963511 | NULL | 2015–01–15 | MK858112 | India | 2017–01–15 |
| JF327392 | Singapore | 2009–01–15 | MK858113 | India | 2017–01–15 |
| JN851113 | Singapore | 2006–01–15 | MK858114 | India | 2017–01–15 |

| Accession no. | Location | Collection date | Accession no. | Location | Collection date |
|---|---|---|---|---|---|
| JN851114 | Singapore | 2007–01–15 | MK858115 | India | 2017–01–15 |
| JN851115 | Singapore | 2007–01–15 | MK858116 | India | 2017–01–15 |
| JN851116 | Singapore | 2007–01–15 | MN018337 | China | 2015–01–15 |
| JN851117 | Singapore | 2007–01–15 | MN018338 | China | 2015–01–15 |
| JN851118 | Singapore | 2008–01–15 | MN018339 | China | 2014–01–15 |
| JN851119 | Singapore | 2008–01–15 | MN018340 | China | 2016–01–15 |
| JN851120 | Singapore | 2007–01–15 | MN018341 | China | 2017–01–15 |
| JN851121 | Singapore | 2008–01–15 | MN018342 | China | 2017–01–15 |
| JN851122 | Singapore | 2008–01–15 | MN018343 | China | 2017–01–15 |
| JN851123 | Singapore | 2004–01–15 | MN018344 | China | 2017–01–15 |
| JN851124 | Singapore | 2005–01–15 | MN018346 | China | 2016–01–15 |
| JN851125 | Singapore | 2005–01–15 | MN018347 | China | 2016–01–15 |
| JN851126 | Singapore | 2005–01–15 | MN018348 | China | 2016–01–15 |
| JN851127 | Singapore | 2004–01–15 | MN018349 | China | 2015–01–15 |
| JN851128 | Singapore | 2006–01–15 | MN018350 | China | 2016–01–15 |
| JN851129 | Singapore | 2006–01–15 | MN018351 | China | 2015–01–15 |
| JN851130 | Singapore | 2005–01–15 | MN018352 | China | 2015–01–15 |
| JN851131 | Singapore | 2005–01–15 | MN018353 | China | 2016–01–15 |
| JQ922549 | India | 1996–01–15 | MN018354 | China | 2017–01–15 |
| JQ922551 | India | 2005–01–15 | MN018355 | China | 2017–01–15 |
| JQ955623 | India | 2009–01–15 | MN018356 | China | 2014–01–15 |
| JQ955624 | India | 2011–01–15 | MN018357 | China | 2014–01–15 |
| JX470186 | China | 2010–01–15 | MN018358 | China | 2016–01–15 |
| JX475906 | India | 2009–01–15 | MN018359 | China | 2017–01–15 |
| KC131142 | China | 2012–09–01 | MN018360 | China | 2017–01–15 |
| KC762655 | Indonesia | 2007–09–27 | MN018361 | China | 2017–01–15 |
| KC762656 | Indonesia | 2007–12–11 | MN018363 | China | 2016–01–15 |
| KC762657 | Indonesia | 2008–04–23 | MN018364 | China | 2016–01–15 |
| KC762658 | Indonesia | 2007–08–14 | MN018365 | China | 2015–01–15 |
| KC762659 | Indonesia | 2008–04–15 | MN253134 | India | 2017–01–15 |
| KC762660 | Indonesia | 2007–11–21 | MN272404 | Reunion | 2018–01–15 |
| KC762661 | Indonesia | 2007–09–24 | MN272405 | Seychelles | 2016–01–15 |
| KC762662 | Indonesia | 2007–11–21 | MN294937 | Saudi Arabia | 2016–01–15 |
| KC762663 | Indonesia | 2008–02–20 | MN328061 | Bangladesh | 2019–01–15 |
| KC762664 | Indonesia | 2008–03–28 | MN335244 | Kenya | 2017–01–15 |
| KC762665 | Indonesia | 2007–07–06 | MN335245 | Kenya | 2017–01–15 |
| KC762666 | Indonesia | 2008–02–15 | MN335246 | Kenya | 2017–01–15 |
| KC762667 | Indonesia | 2008–04–12 | MN335247 | Kenya | 2017–01–15 |
| KC762668 | Indonesia | 2008–03–11 | MN566109 | New Caledonia | 2017–01–15 |
| KC762669 | Indonesia | 2007–07–17 | MN566110 | New Caledonia | 2017–01–15 |
| KC762670 | Indonesia | 2007–12–11 | MN566111 | New Caledonia | 2018–01–15 |
| KC762671 | Indonesia | 2008–02–19 | MN566112 | New Caledonia | 2018–01–15 |
| KC762672 | Indonesia | 2008–04–09 | MN577545 | Sri Lanka | 2017–01–15 |
| KC762673 | Indonesia | 2008–03–11 | MN577546 | Sri Lanka | 2017–01–15 |
| KC762674 | Indonesia | 2008–03–11 | MN577547 | Sri Lanka | 2017–01–15 |
| KC762675 | Indonesia | 2008–03–08 | MN577548 | Sri Lanka | 2017–01–15 |
| KC762676 | Indonesia | 2007–07–17 | MN577549 | Sri Lanka | 2017–01–15 |
| KC762677 | Indonesia | 2008–02–15 | MN577551 | Kenya | 2013–01–15 |
| KC762678 | Indonesia | 2010–03–04 | MN577552 | Kenya | 2014–01–15 |
| KC762679 | Indonesia | 2010–03–29 | MN577553 | Kenya | 2017–01–15 |
| KC762680 | Indonesia | 2010–04–05 | MN577554 | Kenya | 2017–01–15 |
| KC964093 | China | 2001–01–15 | MN577555 | Kenya | 2017–01–15 |
| KC964094 | China | 1993–01–15 | MN577556 | Kenya | 2017–01–15 |
| KF041232 | Pakistan | 2011–01–15 | MN577557 | Kenya | 2017–01–15 |
| KF041233 | Pakistan | 2011–01–15 | MN577558 | Kenya | 2017–01–15 |
| KF041234 | Pakistan | 2011–01–15 | MN577559 | Kenya | 2013–01–15 |
| KF041235 | Pakistan | 2009–01–15 | MN577560 | Kenya | 2013–01–15 |
| KF041236 | Pakistan | 2008–01–15 | MN577561 | Kenya | 2013–01–15 |
| KF041237 | Pakistan | 2009–01–15 | MN577562 | Kenya | 2013–01–15 |
| KF360005 | Pakistan | 2010–11–01 | MN577563 | Kenya | 2013–01–15 |
| KF479233 | China | 2013–01–04 | MN577564 | Kenya | 2014–01–15 |
| KF744397 | Philippines | 2001–01–15 | MN577565 | Kenya | 2014–01–15 |
| KF744398 | Philippines | 2005–01–15 | MN720746 | France | 2014–01–15 |
| KJ010185 | Pakistan | 2011–10–20 | MN923107 | China | 2019–01–15 |
| KJ010186 | Pakistan | 2013–10–02 | MN923108 | China | 2019–01–15 |
| KJ701507 | Pakistan | 2013–08–27 | MN923109 | China | 2019–01–15 |
| KJ830750 | Saudi Arabia | 2014–01–13 | MN923110 | China | 2019–01–15 |
| KM217156 | Pakistan | 2011–08–01 | MN923111 | China | 2019–01–15 |
| KM217157 | Pakistan | 2011–08–01 | MN923112 | China | 2019–01–15 |

| Accession no. | Location | Collection date | Accession no. | Location | Collection date |
|---|---|---|---|---|---|
| KM217158 | Pakistan | 2013–08–01 | MN923116 | China | 2019–01–15 |
| KM279513 | Singapore | 2011–01–15 | MN923117 | China | 2019–01–15 |
| KM279514 | Singapore | 2011–01–15 | MN923118 | China | 2019–01–15 |
| KM279515 | Singapore | 2011–01–15 | MN923119 | China | 2019–01–15 |
| KM279516 | Singapore | 2011–01–15 | MN923121 | China | 2019–01–15 |
| KM279517 | Singapore | 2011–01–15 | MN944002 | China | 2019–01–15 |
| KM279518 | Singapore | 2011–01–15 | MN952966 | China | 2015–01–15 |
| KM279519 | Singapore | 2011–01–15 | MN952967 | China | 2015–01–15 |
| KM279520 | Singapore | 2011–01–15 | MN982899 | Australia | 2019–01–15 |
| KM279521 | Singapore | 2011–01–15 | MT006136 | Sri Lanka | 2017–01–15 |
| KM279522 | Singapore | 2011–01–15 | MT006137 | Sri Lanka | 2017–01–15 |
| KM279523 | Singapore | 2011–01–15 | MT006138 | Sri Lanka | 2017–01–15 |
| KM279524 | Singapore | 2011–01–15 | MT006139 | Sri Lanka | 2017–01–15 |
| KM279525 | Singapore | 2011–01–15 | MT006140 | Sri Lanka | 2017–01–15 |
| KM279526 | Singapore | 2011–01–15 | MT006141 | Sri Lanka | 2017–01–15 |
| KM279527 | Singapore | 2011–01–15 | MT006142 | Sri Lanka | 2018–01–15 |
| KM279528 | Singapore | 2012–01–15 | MT006143 | Sri Lanka | 2018–01–15 |
| KM279529 | Singapore | 2012–01–15 | MT006144 | Sri Lanka | 2018–01–15 |
| KM279530 | Singapore | 2012–01–15 | MT006145 | Sri Lanka | 2018–01–15 |
| KM279531 | Singapore | 2012–01–15 | MT006146 | Sri Lanka | 2017–01–15 |
| KM279532 | Singapore | 2012–01–15 | MT006148 | Sri Lanka | 2017–01–15 |
| KM279533 | Singapore | 2012–01–15 | MT006149 | Sri Lanka | 2018–01–15 |
| KM279534 | Singapore | 2012–01–15 | MT006150 | Sri Lanka | 2017–01–15 |
| KM279535 | Singapore | 2012–01–15 | MT006153 | Sri Lanka | 2017–01–15 |
| KM279536 | Singapore | 2012–01–15 | MT006155 | Sri Lanka | 2017–01–15 |
| KM279537 | Singapore | 2012–01–15 | MT006157 | Sri Lanka | 2017–01–15 |
| KM279538 | Singapore | 2012–01–15 | MT006159 | Sri Lanka | 2017–01–15 |
| KM279539 | Singapore | 2012–01–15 | MT006160 | Sri Lanka | 2017–01–15 |
| KM279540 | Singapore | 2012–01–15 | MT006161 | Sri Lanka | 2017–01–15 |
| KM279541 | Singapore | 2012–01–15 | MT006162 | Sri Lanka | 2017–01–15 |
| KM279542 | Singapore | 2012–01–15 | MT006163 | Sri Lanka | 2018–01–15 |
| KM279543 | Singapore | 2012–01–15 | MT006164 | Sri Lanka | 2018–01–15 |
| KM279544 | Singapore | 2012–01–15 | MT006165 | Sri Lanka | 2018–01–15 |
| KM279545 | Singapore | 2012–01–15 | MT006167 | Sri Lanka | 2017–01–15 |
| KM279546 | Singapore | 2012–01–15 | MT006168 | Sri Lanka | 2018–01–15 |
| KM279547 | Singapore | 2012–01–15 | MT006170 | Sri Lanka | 2018–01–15 |
| KM279548 | Singapore | 2012–01–15 | MT006171 | Sri Lanka | 2018–01–15 |
| KM279549 | Singapore | 2012–01–15 | MT006172 | Sri Lanka | 2018–01–15 |
| KM279550 | Singapore | 2012–01–15 | MT006173 | Sri Lanka | 2018–01–15 |
| KM279551 | Singapore | 2012–01–15 | MT006174 | Sri Lanka | 2018–01–15 |
| KM279552 | Singapore | 2012–01–15 | MT006175 | Sri Lanka | 2018–01–15 |
| KM279553 | Singapore | 2012–01–15 | MT006176 | Sri Lanka | 2018–01–15 |
| KM279554 | Singapore | 2012–01–15 | MT006177 | Sri Lanka | 2018–01–15 |
| KM279555 | Singapore | 2012–01–15 | MT006178 | Sri Lanka | 2018–01–15 |
| KM279556 | Singapore | 2012–01–15 | MT006179 | Sri Lanka | 2018–01–15 |
| KM279557 | Singapore | 2012–01–15 | MT006180 | Sri Lanka | 2018–01–15 |
| KM279558 | Singapore | 2012–01–15 | MT006181 | Sri Lanka | 2018–01–15 |
| KM279559 | Singapore | 2012–01–15 | MT006184 | Sri Lanka | 2018–01–15 |
| KM279560 | Singapore | 2012–01–15 | MT006185 | Sri Lanka | 2018–01–15 |
| KM279561 | Singapore | 2012–01–15 | MT006186 | Sri Lanka | 2018–01–15 |
| KM279562 | Singapore | 2012–01–15 | MT180479 | Sri Lanka | 2017–01–15 |
| KM279563 | Singapore | 2012–01–15 | MT261956 | Burkina Faso | 2017–01–15 |
| KM279564 | Singapore | 2012–01–15 | MT261957 | Burkina Faso | 2017–01–15 |
| KM279565 | Singapore | 2012–01–15 | MT261958 | Burkina Faso | 2017–01–15 |
| KM279566 | Singapore | 2012–01–15 | MT261959 | Burkina Faso | 2017–01–15 |
| KM279567 | Singapore | 2012–01–15 | MT261960 | Burkina Faso | 2017–01–15 |
| KM279568 | Singapore | 2012–01–15 | MT261961 | Burkina Faso | 2017–01–15 |
| KM279569 | Singapore | 2012–01–15 | MT261962 | Burkina Faso | 2017–01–15 |
| KM279570 | Singapore | 2012–01–15 | MT261963 | Burkina Faso | 2017–01–15 |
| KM279571 | Singapore | 2012–01–15 | MT261964 | Burkina Faso | 2017–01–15 |
| KM279572 | Singapore | 2012–01–15 | MT261965 | Burkina Faso | 2017–01–15 |
| KM279573 | Singapore | 2012–01–15 | MT261966 | Burkina Faso | 2017–01–15 |
| KM279574 | Singapore | 2012–01–15 | MT261967 | Burkina Faso | 2017–01–15 |
| KM279575 | Singapore | 2012–01–15 | MT261968 | Burkina Faso | 2017–01–15 |
| KM279576 | Singapore | 2012–01–15 | MT261969 | Burkina Faso | 2017–01–15 |
| KM279577 | Singapore | 2012–01–15 | MT261970 | Burkina Faso | 2017–01–15 |
| KM279578 | Singapore | 2012–01–15 | MT261971 | Burkina Faso | 2017–01–15 |
| KM279579 | Singapore | 2012–01–15 | MT754367 | China | 2019–01–15 |
| KM279580 | Singapore | 2013–01–15 | MT754368 | China | 2019–01–15 |

| Accession no. | Location | Collection date | Accession no. | Location | Collection date |
|---|---|---|---|---|---|
| KM279581 | Singapore | 2011–01–15 | MT754369 | China | 2019–01–15 |
| KM279582 | Singapore | 2011–01–15 | MT754370 | China | 2019–01–15 |
| KM279583 | Singapore | 2011–01–15 | MT754371 | China | 2019–01–15 |
| KM279584 | Singapore | 2011–01–15 | MT832053 | Philippines | 2014–01–15 |
| KM279585 | Singapore | 2011–01–15 | MT832054 | Philippines | 2014–01–15 |
| KM279586 | Singapore | 2011–01–15 | MT832055 | Philippines | 2013–01–15 |
| KM279587 | Singapore | 2012–01–15 | MT832056 | Philippines | 2013–01–15 |
| KM279588 | Singapore | 2012–01–15 | MT832057 | Philippines | 2015–01–15 |
| KM279589 | Singapore | 2012–01–15 | MT832058 | Philippines | 2015–01–15 |
| KM279590 | Singapore | 2012–01–15 | MT832059 | Philippines | 2014–01–15 |
| KM279591 | Singapore | 2012–01–15 | MT832060 | Philippines | 2014–01–15 |
| KM279592 | Singapore | 2012–01–15 | MT832061 | Philippines | 2013–01–15 |
| KM279593 | Singapore | 2012–01–15 | MT832062 | Philippines | 2013–01–15 |
| KM279594 | Singapore | 2012–01–15 | MT832063 | Philippines | 2013–01–15 |
| KM279595 | Singapore | 2012–01–15 | MT832064 | Philippines | 2013–01–15 |
| KM279596 | Singapore | 2012–01–15 | MT832065 | Philippines | 2014–01–15 |
| KM279597 | Singapore | 2012–01–15 | MT832066 | Philippines | 2014–01–15 |
| KM279598 | Singapore | 2012–01–15 | MT832067 | Philippines | 2013–01–15 |
| KM279599 | Singapore | 2012–01–15 | MT832068 | Philippines | 2013–01–15 |
| KM279600 | Singapore | 2012–01–15 | MT832069 | Philippines | 2013–01–15 |
| KM279601 | Singapore | 2012–01–15 | MT832070 | Philippines | 2013–01–15 |
| KM279602 | Singapore | 2008–01–15 | MT832071 | Philippines | 2013–01–15 |
| KM279603 | Singapore | 2007–01–15 | MT832072 | Philippines | 2013–01–15 |
| KM279604 | Singapore | 2008–01–15 | MT832073 | Philippines | 2013–01–15 |
| KM279605 | Singapore | 2009–01–15 | MT832074 | Philippines | 2013–01–15 |
| KM279606 | Singapore | 2010–01–15 | MT832075 | Philippines | 2013–01–15 |
| KM279607 | Singapore | 2010–01–15 | MT832076 | Philippines | 2013–01–15 |
| KM279608 | Singapore | 2010–01–15 | MT832077 | Philippines | 2013–01–15 |
| KM279609 | Singapore | 2009–01–15 | MT832078 | Philippines | 2013–01–15 |
| KM279610 | Singapore | 2010–01–15 | MT832079 | Philippines | 2013–01–15 |
| KP012546 | China | 2014–01–15 | MT832080 | Philippines | 2013–01–15 |
| KP723478 | China | 2014–01–15 | MT921570 | Australia | 2013–01–15 |
| KP723479 | China | 2010–01–15 | MT921571 | Australia | 2015–01–15 |
| KR779782 | Singapore | 2007–01–15 | MT921572 | Australia | 2000–01–15 |
| KR779786 | Singapore | 2013–01–15 | MT921573 | Australia | 2004–01–15 |
| KR920365 | China | 2015–01–15 | MT980927 | Mauritania | 2017–01–15 |
| KT187553 | China | 2014–01–15 | MT981011 | Senegal | 2018–01–15 |
| KT187554 | China | 2014–01–15 | MT981085 | Mauritania | 2018–01–15 |
| KT187555 | China | 2014–01–15 | MT981148 | Senegal | 2019–01–15 |
| KT187556 | China | 2014–01–15 | MT982126 | Burkina Faso | 2019–01–15 |
| KT187557 | China | 2014–01–15 | MT982148 | Burkina Faso | 2019–01–15 |
| KT187558 | China | 2014–01–15 | MT982169 | Côte d’Ivoire | 2017–01–15 |
| KU365901 | Taiwan | 2015–01–15 | MT982731 | Burkina Faso | 2016–01–15 |
| KU365902 | Taiwan | 2015–01–15 | MW186239 | Singapore | 2019–01–15 |
| KU365903 | Taiwan | 2015–01–15 | MW186240 | Singapore | 2019–01–15 |
| KU509268 | Indonesia | 2009–01–15 | MW288024 | Senegal | 2018–01–15 |
| KU509269 | Philippines | 2009–01–15 | MW288029 | Senegal | 2018–01–15 |
| KU509270 | Germany | 2012–01–15 | MW288030 | Senegal | 2018–01–15 |
| KU509271 | India | 2006–01–15 | MW288034 | Senegal | 2018–01–15 |
| KU509272 | Thailand | 2009–01–15 | MW295816 | China | 2020–01–15 |
| KU509274 | Philippines | 2010–01–15 | MW295818 | China | 2020–01–15 |
| KU509275 | Philippines | 2008–01–15 | MW345921 | China | 2020–01–15 |
| KU509276 | Philippines | 2008–01–15 | MW387614 | China | 2009–01–15 |
| KU509277 | Philippines | 2010–01–15 | MW481670 | Angola | 2019–01–15 |
| KU517845 | Papua New Guinea | 2013–01–15 | MW481671 | Angola | 2018–01–15 |
| KU517846 | Indonesia | 2014–01–15 | MW481672 | Angola | 2018–01–15 |
| KU517847 | Philippines | 2015–01–15 | MW481673 | Angola | 2018–01–15 |
| KU666944 | Malaysia | 2014–01–15 | MW481674 | Angola | 2018–01–15 |
| KU666945 | Malaysia | 2014–01–15 | MW481675 | Angola | 2018–01–15 |
| KU666946 | Malaysia | 2014–01–15 | MW481676 | Angola | 2018–01–15 |
| KU666947 | Malaysia | 2014–01–15 | MW481677 | Angola | 2018–01–15 |
| KU666948 | Malaysia | 2014–01–15 | MW481678 | Angola | 2018–01–15 |
| KU666949 | Malaysia | 2014–01–15 | MW481679 | Angola | 2018–01–15 |
| KU948303 | Singapore | 2016–01–15 | MW481680 | Angola | 2018–01–15 |
| KX225485 | China | 2015–01–15 | MW481681 | Angola | 2018–01–15 |
| KX225486 | China | 2015–01–15 | MW481682 | Angola | 2018–01–15 |
| KX372564 | Australia | 2015–01–15 | MW481683 | Angola | 2018–01–15 |
| KX380807 | Singapore | 2012–01–15 | MW481684 | Angola | 2018–01–15 |
| KX380808 | Singapore | 2012–01–15 | MW481685 | Angola | 2018–01–15 |

| Accession no. | Location | Collection date | Accession no. | Location | Collection date |
|---|---|---|---|---|---|
| KX380809 | Singapore | 2012–01–15 | MW481686 | Angola | 2018–01–15 |
| KX380810 | Singapore | 2012–01–15 | MW481687 | Angola | 2018–01–15 |
| KX380811 | Singapore | 2012–01–15 | MW481688 | Angola | 2018–01–15 |
| KX380812 | Singapore | 2012–01–15 | MW481689 | Angola | 2018–01–15 |
| KX380813 | Singapore | 2012–01–15 | MW481690 | Angola | 2018–01–15 |
| KX380814 | Singapore | 2012–01–15 | MW481691 | Angola | 2018–01–15 |
| KX380815 | Singapore | 2012–01–15 | MW481692 | Angola | 2018–01–15 |
| KX380816 | Singapore | 2012–01–15 | MW481693 | Angola | 2018–01–15 |
| KX380817 | Singapore | 2012–01–15 | MW481694 | Angola | 2018–01–15 |
| KX380818 | Singapore | 2012–01–15 | MW512341 | Singapore | 2004–01–15 |
| KX380819 | Singapore | 2012–01–15 | MW512342 | Singapore | 2007–01–15 |
| KX380820 | Singapore | 2012–01–15 | MW512343 | Singapore | 2007–01–15 |
| KX380821 | Singapore | 2012–01–15 | MW512344 | Singapore | 2007–01–15 |
| KX380822 | Singapore | 2012–01–15 | MW512345 | Singapore | 2007–01–15 |
| KX380823 | Singapore | 2012–01–15 | MW512346 | Singapore | 2007–01–15 |
| KX380824 | Singapore | 2012–01–15 | MW512347 | Singapore | 2007–01–15 |
| KX380825 | Singapore | 2013–01–15 | MW512348 | Singapore | 2007–01–15 |
| KX380826 | Singapore | 2013–01–15 | MW512349 | Singapore | 2007–01–15 |
| KX380827 | Singapore | 2013–01–15 | MW512350 | Singapore | 2008–01–15 |
| KX380828 | Singapore | 2013–01–15 | MW512351 | Singapore | 2008–01–15 |
| KX380829 | Singapore | 2013–01–15 | MW512352 | Singapore | 2008–01–15 |
| KX380830 | Singapore | 2013–01–15 | MW512353 | Singapore | 2008–01–15 |
| KX380831 | Singapore | 2013–01–15 | MW512354 | Singapore | 2009–01–15 |
| KX380832 | Singapore | 2013–01–15 | MW512355 | Singapore | 2009–01–15 |
| KX380833 | Singapore | 2013–01–15 | MW512356 | Singapore | 2009–01–15 |
| KX380834 | Singapore | 2013–01–15 | MW512357 | Singapore | 2010–01–15 |
| KX380835 | Singapore | 2013–01–15 | MW512358 | Singapore | 2010–01–15 |
| KX380836 | Singapore | 2013–01–15 | MW512359 | Singapore | 2010–01–15 |
| KX380837 | Singapore | 2013–01–15 | MW512360 | Singapore | 2010–01–15 |
| KX380838 | Singapore | 2013–01–15 | MW512361 | Singapore | 2010–01–15 |
| KX452015 | Malaysia | 2014–01–15 | MW512362 | Singapore | 2011–01–15 |
| KX452016 | Malaysia | 2014–01–15 | MW512363 | Singapore | 2011–01–15 |
| KX452017 | Malaysia | 2014–01–15 | MW512364 | Singapore | 2011–01–15 |
| KX452018 | Malaysia | 2014–01–15 | MW512365 | Singapore | 2011–01–15 |
| KX452019 | Malaysia | 2014–01–15 | MW512366 | Singapore | 2011–01–15 |
| KX452020 | Malaysia | 2014–01–15 | MW512367 | Singapore | 2011–01–15 |
| KX452021 | Malaysia | 2014–01–15 | MW512368 | Singapore | 2011–01–15 |
| KX452022 | Malaysia | 2014–01–15 | MW512369 | Singapore | 2011–01–15 |
| KX452023 | Malaysia | 2014–01–15 | MW512370 | Singapore | 2012–01–15 |
| KX452024 | Malaysia | 2014–01–15 | MW512371 | Singapore | 2012–01–15 |
| KX452025 | Malaysia | 2014–01–15 | MW512372 | Singapore | 2012–01–15 |
| KX452026 | Malaysia | 2014–01–15 | MW512373 | Singapore | 2012–01–15 |
| KX452027 | Malaysia | 2014–01–15 | MW512374 | Singapore | 2012–01–15 |
| KX452028 | Malaysia | 2014–01–15 | MW512375 | Singapore | 2012–01–15 |
| KX452029 | Malaysia | 2014–01–15 | MW512376 | Singapore | 2012–01–15 |
| KX452030 | Malaysia | 2014–01–15 | MW512377 | Singapore | 2012–01–15 |
| KX452031 | Malaysia | 2014–01–15 | MW512378 | Singapore | 2012–01–15 |
| KX452032 | Malaysia | 2014–01–15 | MW512379 | Singapore | 2012–01–15 |
| KX452033 | Malaysia | 2014–01–15 | MW512380 | Singapore | 2012–01–15 |
| KX452034 | Malaysia | 2014–01–15 | MW512381 | Singapore | 2013–01–15 |
| KX452035 | Malaysia | 2014–01–15 | MW512382 | Singapore | 2013–01–15 |
| KX452036 | Malaysia | 2014–01–15 | MW512383 | Singapore | 2013–01–15 |
| KX452037 | Malaysia | 2014–01–15 | MW512384 | Singapore | 2013–01–15 |
| KX452038 | Malaysia | 2014–01–15 | MW512385 | Singapore | 2013–01–15 |
| KX452039 | Malaysia | 2014–01–15 | MW512386 | Singapore | 2013–01–15 |
| KX452040 | Malaysia | 2014–01–15 | MW512387 | Singapore | 2013–01–15 |
| KX452041 | Malaysia | 2014–01–15 | MW512388 | Singapore | 2013–01–15 |
| KX452042 | Malaysia | 2014–01–15 | MW512389 | Singapore | 2013–01–15 |
| KX452043 | Malaysia | 2014–01–15 | MW512390 | Singapore | 2013–01–15 |
| KX452044 | Malaysia | 2014–01–15 | MW512391 | Singapore | 2013–01–15 |
| KX452045 | Malaysia | 2014–01–15 | MW512392 | Singapore | 2013–01–15 |
| KX452046 | Malaysia | 2014–01–15 | MW512393 | Singapore | 2013–01–15 |
| KX452047 | Malaysia | 2014–01–15 | MW512394 | Singapore | 2013–01–15 |
| KX452048 | Malaysia | 2014–01–15 | MW512395 | Singapore | 2013–01–15 |
| KX452049 | Malaysia | 2014–01–15 | MW512396 | Singapore | 2013–01–15 |
| KX621245 | China | 2015–01–15 | MW512397 | Singapore | 2013–01–15 |
| KX621246 | China | 2015–01–15 | MW512398 | Singapore | 2013–01–15 |
| KX621247 | China | 2015–01–15 | MW512399 | Singapore | 2013–01–15 |
| KX621248 | China | 2015–01–15 | MW512400 | Singapore | 2013–01–15 |

| Accession no. | Location | Collection date | Accession no. | Location | Collection date |
|---|---|---|---|---|---|
| KX655786 | China | 2015–01–15 | MW512401 | Singapore | 2013–01–15 |
| KX655787 | China | 2015–01–15 | MW512402 | Singapore | 2013–01–15 |
| KX655788 | China | 2015–01–15 | MW512403 | Singapore | 2013–01–15 |
| KY427084 | India | 2010–01–15 | MW512404 | Singapore | 2013–01–15 |
| KY427085 | India | 2011–01–15 | MW512405 | Singapore | 2013–01–15 |
| KY627762 | Burkina Faso | 2016–01–15 | MW512406 | Singapore | 2013–01–15 |
| KY627763 | Burkina Faso | 2016–01–15 | MW512407 | Singapore | 2013–01–15 |
| KY672950 | China | 2015–01–15 | MW512408 | Singapore | 2013–01–15 |
| KY672951 | China | 2015–01–15 | MW512409 | Singapore | 2013–01–15 |
| KY672952 | China | 2015–01–15 | MW512410 | Singapore | 2014–01–15 |
| KY672953 | China | 2015–01–15 | MW512411 | Singapore | 2014–01–15 |
| KY672954 | China | 2015–01–15 | MW512412 | Singapore | 2014–01–15 |
| KY794785 | Papua New Guinea | 2010–01–15 | MW512413 | Singapore | 2014–01–15 |
| KY882458 | China | 2017–01–15 | MW512414 | Singapore | 2014–01–15 |
| KY921904 | Singapore | 2014–01–15 | MW512415 | Singapore | 2014–01–15 |
| KY921905 | Singapore | 2015–01–15 | MW512416 | Singapore | 2014–01–15 |
| KY937185 | China | 2015–01–15 | MW512417 | Singapore | 2014–01–15 |
| KY937186 | China | 2015–01–15 | MW512418 | Singapore | 2014–01–15 |
| KY937187 | China | 2015–01–15 | MW512419 | Singapore | 2014–01–15 |
| KY937188 | China | 2015–01–15 | MW512420 | Singapore | 2014–01–15 |
| KY937189 | China | 2015–01–15 | MW512421 | Singapore | 2014–01–15 |
| KY937190 | China | 2015–01–15 | MW512422 | Singapore | 2014–01–15 |
| LC111438 | East Timor | 2005–01–15 | MW512423 | Singapore | 2014–01–15 |
| LC121816 | Ethiopia | 2016–01–01 | MW512424 | Singapore | 2014–01–15 |
| LC367234 | India | 2009–01–15 | MW512425 | Singapore | 2014–01–15 |
| LC410189 | Thailand | 2016–01–15 | MW512426 | Singapore | 2014–01–15 |
| LC410190 | Thailand | 2016–01–15 | MW512427 | Singapore | 2014–01–15 |
| LC410191 | Thailand | 2017–01–15 | MW512428 | Singapore | 2014–01–15 |
| LC436669 | Bangladesh | 2017–01–15 | MW512429 | Singapore | 2014–01–15 |
| LC436670 | Bangladesh | 2017–01–15 | MW512430 | Singapore | 2014–01–15 |
| LC436671 | Bangladesh | 2017–01–15 | MW512431 | Singapore | 2014–01–15 |
| LC436672 | Bangladesh | 2017–01–15 | MW512432 | Singapore | 2014–01–15 |
| LC436673 | Bangladesh | 2017–01–15 | MW512433 | Singapore | 2014–01–15 |
| LC436674 | Bangladesh | 2017–01–15 | MW512434 | Singapore | 2014–01–15 |
| LC436675 | Bangladesh | 2017–01–15 | MW512435 | Singapore | 2015–01–15 |
| LC666718 | Ghana | 2017–01–15 | MW512436 | Singapore | 2015–01–15 |
| LC666719 | Ghana | 2017–01–15 | MW512437 | Singapore | 2015–01–15 |
| MF004385 | France | 2014–01–15 | MW512438 | Singapore | 2015–01–15 |
| MF043956 | China | 2016–01–15 | MW512439 | Singapore | 2015–01–15 |
| MF156233 | China | 2015–01–15 | MW512440 | Singapore | 2015–01–15 |
| MF156234 | China | 2015–01–15 | MW512441 | Singapore | 2015–01–15 |
| MF156235 | China | 2015–01–15 | MW512442 | Singapore | 2015–01–15 |
| MF156236 | China | 2015–01–15 | MW512443 | Singapore | 2015–01–15 |
| MF156237 | China | 2015–01–15 | MW512444 | Singapore | 2015–01–15 |
| MF156238 | China | 2015–01–15 | MW512445 | Singapore | 2015–01–15 |
| MF156239 | China | 2015–01–15 | MW512446 | Singapore | 2015–01–15 |
| MF156240 | China | 2015–01–15 | MW512447 | Singapore | 2015–01–15 |
| MF156241 | China | 2015–01–15 | MW512448 | Singapore | 2015–01–15 |
| MF156242 | China | 2015–01–15 | MW512449 | Singapore | 2016–01–15 |
| MF156243 | China | 2015–01–15 | MW512450 | Singapore | 2016–01–15 |
| MF156244 | China | 2015–01–15 | MW512451 | Singapore | 2016–01–15 |
| MF156245 | China | 2015–01–15 | MW512452 | Singapore | 2016–01–15 |
| MF156246 | China | 2015–01–15 | MW512453 | Singapore | 2016–01–15 |
| MF156247 | China | 2015–01–15 | MW512454 | Singapore | 2016–01–15 |
| MF156248 | China | 2015–01–15 | MW512455 | Singapore | 2016–01–15 |
| MF314189 | Singapore | 2016–01–15 | MW512456 | Singapore | 2016–01–15 |
| MF940236 | China | 2015–01–15 | MW512457 | Singapore | 2016–01–15 |
| MF940237 | China | 2015–01–15 | MW512458 | Singapore | 2016–01–15 |
| MF940238 | China | 2015–01–15 | MW512459 | Singapore | 2016–01–15 |
| MF940239 | China | 2015–01–15 | MW512460 | Singapore | 2016–01–15 |
| MF940240 | China | 2015–01–15 | MW512461 | Singapore | 2016–01–15 |
| MF940241 | China | 2015–01–15 | MW512462 | Singapore | 2016–01–15 |
| MF940242 | China | 2015–01–15 | MW512463 | Singapore | 2016–01–15 |
| MF940243 | China | 2015–01–15 | MW512464 | Singapore | 2016–01–15 |
| MF940244 | China | 2015–01–15 | MW512465 | Singapore | 2016–01–15 |
| MF940245 | China | 2015–01–15 | MW512466 | Singapore | 2016–01–15 |
| MF940246 | China | 2015–01–15 | MW512467 | Singapore | 2016–01–15 |
| MF940247 | China | 2015–01–15 | MW512468 | Singapore | 2017–01–15 |
| MF940248 | China | 2015–01–15 | MW512469 | Singapore | 2017–01–15 |

| Accession no. | Location | Collection date | Accession no. | Location | Collection date |
|---|---|---|---|---|---|
| MF940249 | China | 2015–01–15 | MW512470 | Singapore | 2017–01–15 |
| MF940250 | China | 2015–01–15 | MW512471 | Singapore | 2017–01–15 |
| MF940251 | China | 2015–01–15 | MW512472 | Singapore | 2017–01–15 |
| MF940252 | China | 2015–01–15 | MW512473 | Singapore | 2017–01–15 |
| MF940253 | China | 2015–01–15 | MW512474 | Singapore | 2017–01–15 |
| MG189962 | Tanzania | 2014–01–15 | MW512475 | Singapore | 2017–01–15 |
| MG560143 | India | 2014–01–15 | MW512476 | Singapore | 2017–01–15 |
| MG560144 | India | 2014–01–15 | MW512477 | Singapore | 2017–01–15 |
| MG592698 | India | 2016–01–15 | MW512478 | Singapore | 2017–01–15 |
| MG721054 | India | 2016–01–15 | MW512479 | Singapore | 2018–01–15 |
| MG721055 | India | 2016–01–15 | MW512480 | Singapore | 2018–01–15 |
| MG721056 | India | 2016–01–15 | MW512481 | Singapore | 2018–01–15 |
| MG721057 | India | 2016–01–15 | MW512482 | Singapore | 2018–01–15 |
| MG721058 | India | 2016–01–15 | MW512483 | Singapore | 2018–01–15 |
| MG721062 | India | 2016–01–15 | MW512484 | Singapore | 2018–01–15 |
| MG779194 | Kenya | 2017–01–15 | MW512485 | Singapore | 2018–01–15 |
| MG779195 | Kenya | 2017–01–15 | MW512486 | Singapore | 2018–01–15 |
| MG779196 | Kenya | 2017–01–15 | MW512487 | Singapore | 2018–01–15 |
| MG779197 | Kenya | 2017–01–15 | MW512488 | Singapore | 2018–01–15 |
| MG779198 | Kenya | 2017–01–15 | MW512489 | Singapore | 2018–01–15 |
| MG779199 | Kenya | 2017–01–15 | MW512490 | Singapore | 2019–01–15 |
| MG779200 | Kenya | 2017–01–15 | MW512491 | Singapore | 2019–01–15 |
| MG779201 | Kenya | 2017–01–15 | MW512492 | Singapore | 2019–01–15 |
| MG779202 | Kenya | 2017–01–15 | MW512493 | Singapore | 2019–01–15 |
| MG779203 | Kenya | 2017–01–15 | MW512494 | Singapore | 2019–01–15 |
| MH010629 | China | 2017–01–15 | MW512495 | Singapore | 2019–01–15 |
| MH048671 | Malaysia | 2014–01–15 | MW512496 | Singapore | 2019–01–15 |
| MH048672 | Malaysia | 2014–01–15 | MW512497 | Singapore | 2019–01–15 |
| MH048673 | Malaysia | 2014–01–15 | MW512498 | Singapore | 2019–01–15 |
| MH048675 | Malaysia | 2014–01–15 | MW730814 | Nepal | 2017–01–15 |
| MH110564 | China | 2017–01–15 | MW730815 | Nepal | 2017–01–15 |
| MH110565 | China | 2017–01–15 | MW730816 | Nepal | 2017–01–15 |
| MH110566 | China | 2017–01–15 | MW730817 | Nepal | 2017–01–15 |
| MH110567 | China | 2017–01–15 | MW730818 | Nepal | 2017–01–15 |
| MH110568 | China | 2017–01–15 | MW730819 | Nepal | 2017–01–15 |
| MH110569 | China | 2017–01–15 | MW730820 | Nepal | 2017–01–15 |
| MH110570 | China | 2017–01–15 | MW730821 | Nepal | 2017–01–15 |
| MH110571 | China | 2017–01–15 | MW730822 | Nepal | 2017–01–15 |
| MH110572 | China | 2017–01–15 | MW730823 | Nepal | 2017–01–15 |
| MH110573 | China | 2017–01–15 | MW730824 | Nepal | 2017–01–15 |
| MH110574 | China | 2017–01–15 | MW730825 | Nepal | 2017–01–15 |
| MH110575 | China | 2017–01–15 | MW730826 | Nepal | 2017–01–15 |
| MH110576 | China | 2017–01–15 | MW730827 | Nepal | 2017–01–15 |
| MH110577 | China | 2017–01–15 | MW730828 | Nepal | 2017–01–15 |
| MH110578 | China | 2017–01–15 | MW730829 | Nepal | 2017–01–15 |
| MH110579 | China | 2017–01–15 | MW730830 | Nepal | 2017–01–15 |
| MH110580 | China | 2017–01–15 | MW730831 | Nepal | 2017–01–15 |
| MH110581 | China | 2017–01–15 | MW730832 | Nepal | 2017–01–15 |
| MH110582 | China | 2017–01–15 | MW730833 | Nepal | 2017–01–15 |
| MH110583 | China | 2017–01–15 | MW730834 | Nepal | 2017–01–15 |
| MH110584 | China | 2017–01–15 | MW730835 | Nepal | 2017–01–15 |
| MH110585 | China | 2017–01–15 | MW730836 | Nepal | 2017–01–15 |
| MH110586 | China | 2017–01–15 | MW730837 | Nepal | 2017–01–15 |
| MH110587 | China | 2017–01–15 | MW730838 | Nepal | 2017–01–15 |
| MH110588 | China | 2017–01–15 | MW730839 | Nepal | 2017–01–15 |
| MH110589 | China | 2017–01–15 | MW881533 | China | 2021–01–15 |
| MH110590 | China | 2017–01–15 | MW945435 | Viet Nam | 2006–01–15 |
| MH110591 | China | 2017–01–15 | MW946433 | Thailand | 2007–01–15 |
| MH110592 | China | 2017–01–15 | MW946478 | India | 1974–01–15 |
| MH110593 | China | 2017–01–15 | MW946584 | Thailand | 2007–01–15 |
| MH110594 | China | 2017–01–15 | MZ312931 | India | 2017–01–15 |
| MH110595 | China | 2017–01–15 | MZ453006 | China | 2019–01–15 |
| MH110596 | China | 2017–01–15 | MZ453008 | China | 2019–01–15 |
| MH110597 | China | 2017–01–15 | MZ453009 | China | 2019–01–15 |
| MH110598 | China | 2017–01–15 | MZ453010 | China | 2019–01–15 |
| MH110599 | China | 2017–01–15 | MZ453011 | China | 2019–01–15 |
| MH110600 | China | 2017–01–15 | MZ636802 | Thailand | 2019–01–15 |
| MH110601 | China | 2017–01–15 | MZ636803 | Thailand | 2019–01–15 |
| MH110602 | China | 2017–01–15 | MZ636804 | Thailand | 2019–01–15 |

| Accession no. | Location | Collection date | Accession no. | Location | Collection date |
|---|---|---|---|---|---|
| MH110603 | China | 2017–01–15 | MZ636805 | Thailand | 2019–01–15 |
| MH456892 | Kenya | 2014–01–15 | MZ857208 | Kenya | 2013–01–15 |
| MH456893 | Kenya | 2014–01–15 | MZ857209 | Kenya | 2013–01–15 |
| MH456894 | Kenya | 2014–01–15 | MZ857210 | Tanzania | 2014–01–15 |
| MH456895 | Kenya | 2014–01–15 | MZ857211 | Mozambique | 2014–01–15 |
| MH456897 | Kenya | 2014–01–15 | MZ857212 | Tanzania | 2014–01–15 |
| MH456898 | Kenya | 2014–01–15 | MZ857213 | Tanzania | 2014–01–15 |
| MH460898 | Angola | 2018–01–15 | MZ857214 | Tanzania | 2014–01–15 |
| MH488959 | Malaysia | 2014–01–15 | MZ857215 | Somalia | 2015–01–15 |
| MH822939 | India | 2012–01–15 | MZ857218 | Eritrea | 2017–01–15 |
| MH822940 | India | 2014–01–15 | MZ857219 | Somalia | 1996–01–15 |
| MH822941 | India | 2013–01–15 | MZ857220 | Burkina Faso | 2017–01–15 |
| MH822942 | India | 2014–01–15 | MZ857221 | Uganda | 2017–01–15 |
| MH822943 | India | 2014–01–15 | MZ857222 | Djibouti | 2007–01–15 |
| MH822944 | India | 2012–01–15 | OK559627 | China | 2019–01–15 |
| MH822945 | India | 2012–01–15 | OL412740 | Cambodia | 2019–01–15 |
| MH822946 | India | 2012–01–15 | OL414717 | Cambodia | 2019–01–15 |
| MH822947 | India | 2012–01–15 | OL414718 | Cambodia | 2019–01–15 |
| MH822948 | India | 2012–01–15 | OL414722 | Cambodia | 2019–01–15 |
| MH822949 | India | 2014–01–15 | OL414723 | Cambodia | 2019–01–15 |
| MH822950 | India | 2013–01–15 | OL414724 | Cambodia | 2019–01–15 |
| MH822951 | India | 2013–01–15 | OL414725 | Cambodia | 2019–01–15 |
| MH822952 | India | 2014–01–15 | OL414726 | Cambodia | 2019–01–15 |
| MH822953 | India | 2015–01–15 | OL414727 | Cambodia | 2019–01–15 |
| MH822954 | India | 2015–01–15 | OL414730 | Cambodia | 2019–01–15 |
| MH822955 | India | 2014–01–15 | OL414731 | Cambodia | 2020–01–15 |
| MH822956 | India | 2013–01–15 | OL414732 | Cambodia | 2020–01–15 |
| MH823208 | Indonesia | 2014–01–15 | OL414733 | Cambodia | 2020–01–15 |
| MH827525 | China | 2017–01–15 | OL414736 | Cambodia | 2020–01–15 |
| MH827526 | China | 2017–01–15 | OL414737 | Cambodia | 2020–01–15 |
| MH827527 | China | 2017–01–15 | OL414738 | Cambodia | 2019–01–15 |
| MH827528 | China | 2017–01–15 | OL414739 | Cambodia | 2019–01–15 |
| MH827529 | China | 2017–01–15 | OL414740 | Cambodia | 2019–01–15 |
| MH827530 | China | 2017–01–15 | OL414741 | Cambodia | 2019–01–15 |
| MH827533 | China | 2017–01–15 | OL414742 | Cambodia | 2019–01–15 |
| MH827534 | China | 2017–01–15 | OL414745 | Cambodia | 2019–01–15 |
| MH827535 | China | 2017–01–15 | OL414746 | Cambodia | 2019–01–15 |
| MH827536 | China | 2017–01–15 | OL414747 | Cambodia | 2019–01–15 |
| MH827537 | China | 2017–01–15 | OL414749 | Cambodia | 2020–01–15 |
| MH827539 | China | 2017–01–15 | OL414750 | Cambodia | 2020–01–15 |
| MH827540 | China | 2017–01–15 | OL414751 | Cambodia | 2020–01–15 |
| MH827541 | China | 2017–01–15 | OL414752 | Cambodia | 2020–01–15 |
| MH827542 | China | 2017–01–15 | OL414753 | Cambodia | 2020–01–15 |
| MH827543 | China | 2017–01–15 | OL414754 | Cambodia | 2020–01–15 |
| MH827544 | China | 2017–01–15 | OL414755 | Cambodia | 2020–01–15 |
| MH827545 | China | 2017–01–15 | OL414756 | Cambodia | 2020–01–15 |
| MH827546 | China | 2017–01–15 | OL414757 | Cambodia | 2020–01–15 |
| MH827547 | China | 2017–01–15 | OL414758 | Cambodia | 2020–01–15 |
| MH827548 | China | 2017–01–15 | OL414759 | Cambodia | 2019–01–15 |
| MH827549 | China | 2017–01–15 | OL414760 | Cambodia | 2019–01–15 |
| MH827550 | China | 2017–01–15 | OL414762 | Cambodia | 2019–01–15 |
| MH827551 | China | 2017–01–15 | OL414763 | Cambodia | 2019–01–15 |
| MH827552 | China | 2017–01–15 | OL414764 | Cambodia | 2020–01–15 |
| MH827553 | China | 2017–01–15 | OL414765 | Cambodia | 2020–01–15 |
| MH827554 | China | 2017–01–15 | OL420733 | Cambodia | 2019–01–15 |
| MH891768 | India | 2016–01–15 | OL435143 | Cambodia | 2020–01–15 |
| MH891770 | India | 2018–01–15 | OL469512 | Côte d’Ivoire | 2017–01–15 |
| MH891772 | India | 2017–01–15 | OL469513 | Côte d’Ivoire | 2017–01–15 |
| MH985858 | Australia | 2016–01–15 | OM317565 | Cameroon | 2020–01–15 |
| MH985859 | Australia | 2017–01–15 | OM317566 | Cameroon | 2020–01–15 |
| MK411558 | Indonesia | 2016–01–15 | OM349569 | China | 2019–01–15 |
| MK411559 | Indonesia | 2016–01–15 | OM368351 | China | 2019–01–15 |
| MK473384 | Kenya | 2016–01–15 | OM368352 | China | 2018–01–15 |
| MK473385 | Kenya | 2016–01–15 | | | |

*Accession numbers were obtained from GenBank.

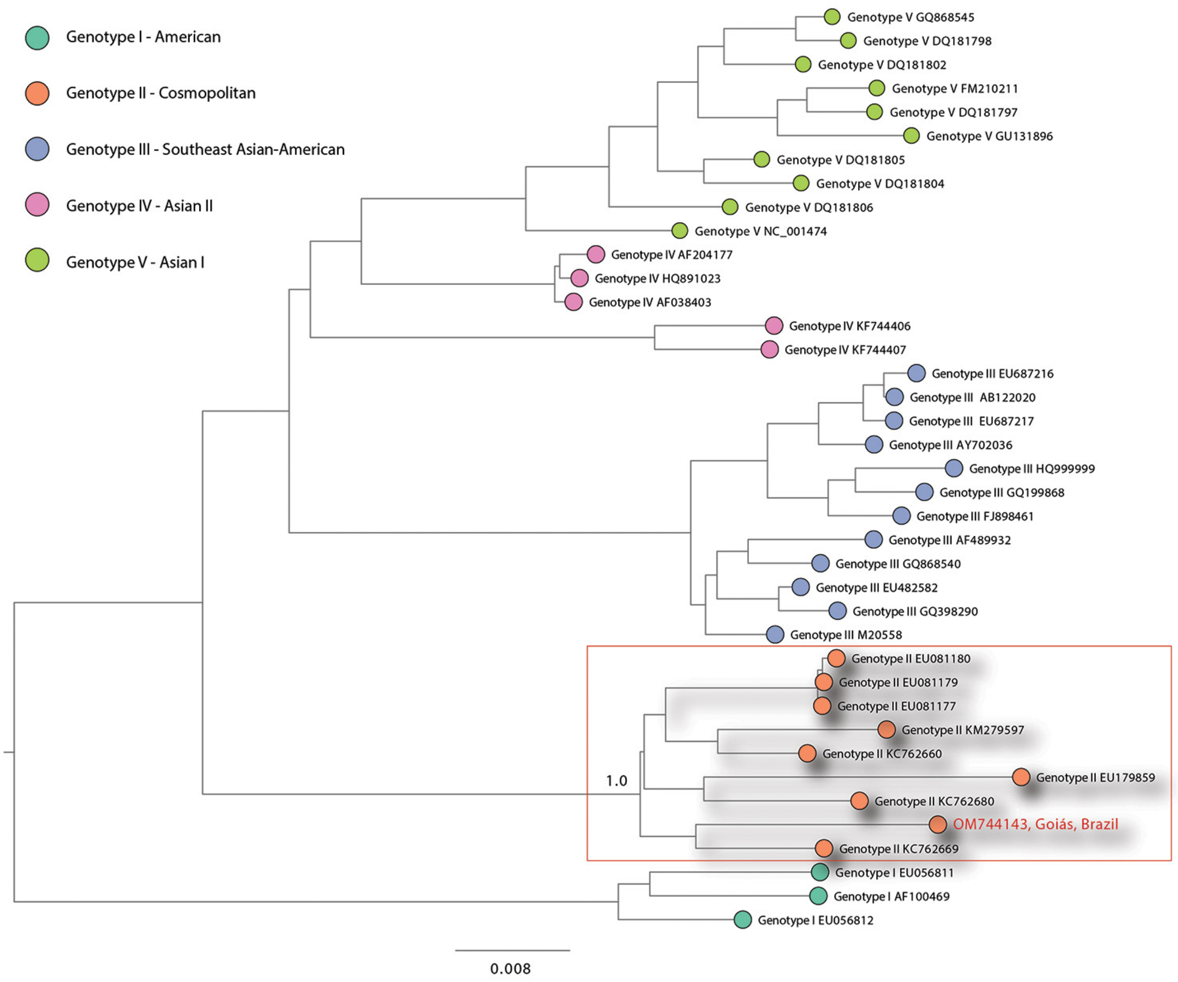


**Appendix Figure 1.** Phylogenetic analysis of DENV-2 cosmopolitan genotype, Brazil. We used the maximum-likelihood method to compare the genome sequence generated in this study with 37 DENV-2 nonsylvatic genotypes retrieved from the National Center for Biotechnology Information database (https://www.ncbi.nlm.nih.gov). Red text indicates sequence described from Brazil. The nonsylvatic genotypes comprised DENV-2 Genotype I–American, DENV-2 Genotype II–cosmopolitan, DENV-2 Genotype III–Southern Asian-American, DENV-2 Genotype IV–Asian II, and DENV-2 Genotype V–Asian I. The boxed region indicates that the strain from Brazil clustered within a clade of cosmopolitan genotypes and had maximum statistical support (bootstrap value = 1.0). Scale bar indicates nucleotide substitutions per site. DENV-2, dengue virus serotype 2.

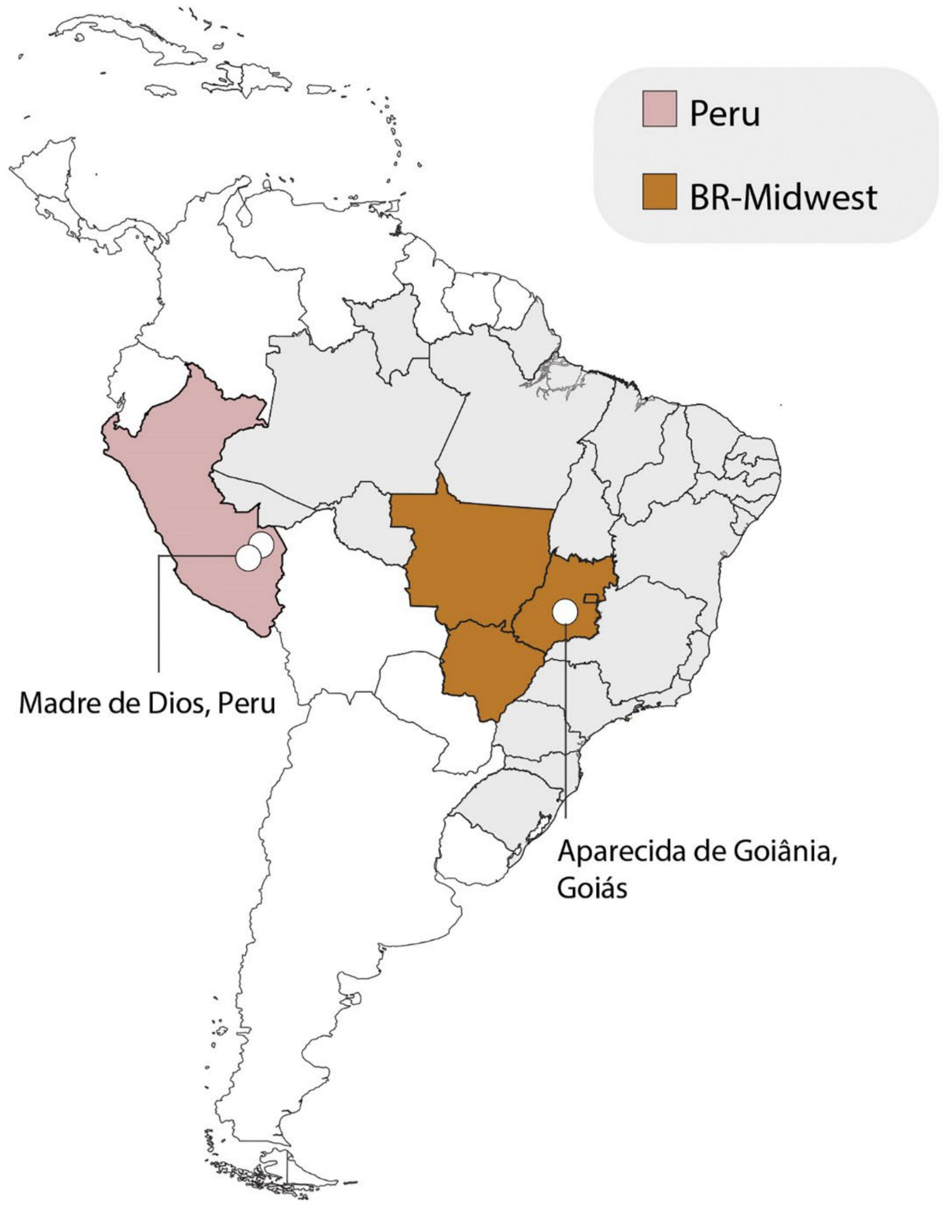


**Appendix Figure 2.** Map of sites in South America from which DENV-2 cosmopolitan genotype has been isolated. Gray area indicates Brazil; brown area indicates midwestern Brazil. Dark pink area indicates Peru. White circles indicate sampling locations of the DENV-2 cosmopolitan genotypes reported in Peru and Brazil. BR, Brazil; DENV-2, dengue virus serotype 2.

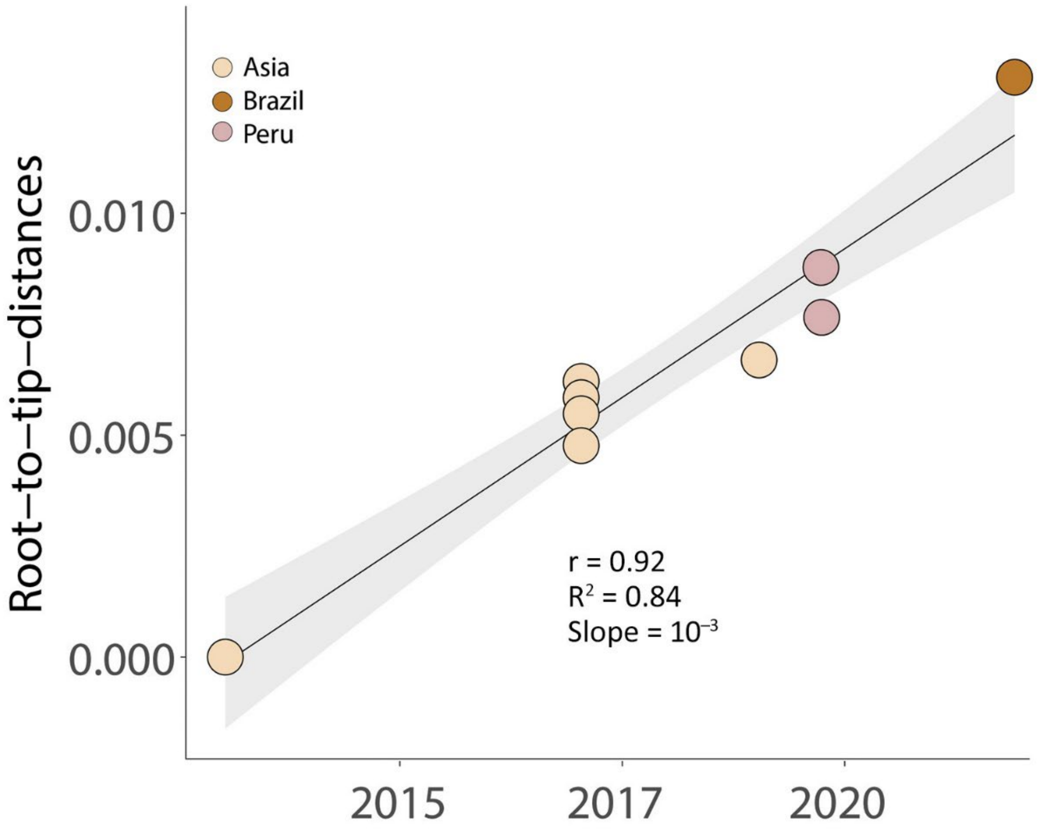


**Appendix Figure 3.** Root-to-tip regression plot of DENV-2 cosmopolitan genotype, Brazil. The root-to-tip genetic distances for genotypes presented in the boxed region of Appendix Figure 1 were plotted against sampling time. The cluster from South America diverged from strains observed in Bangladesh that were collected during 2017–2019, suggesting a complex transmission pattern mediated by transcontinental travel. DENV-2, dengue virus serotype 2.
